# Viewpoints: contrasting opinions in *Neural Development*

**DOI:** 10.1186/1749-8104-4-23

**Published:** 2009-07-06

**Authors:** Andrew Lumsden, Bill Harris, Joshua R Sanes, Rachel Wong

**Affiliations:** 1 MRC Centre for Developmental Neurobiology, King's College London, Guy's Hospital, London SE1 1UL, UK; 2 Department of Physiology, Development and Neuroscience, Cambridge University, Downing St, Cambridge CB2 3DY, UK; 3 Department of Molecular and Cellular Biology and Center for Brain Science, Harvard University, 7 Divinity Ave, Cambridge MA 02138, USA; 4 Department of Biological Structure, University of Washington, HSB G514, Seattle, Washington 98195-7420, USA

We like to think of neurobiology as a field in which clear results lead inexorably to clear conclusions. Sometimes though, the systems we study are so complex that even large data sets are consistent with more than a single interpretation. When the issues are important ones it may be helpful to have proponents of distinct interpretations present their contrasting viewpoints side-by-side, so non-specialists can judge for themselves what all the fuss is about, and whether one view is closer to the mark than the others. In some cases, these pieces might also be didactically useful, for example in neuroscience courses that aim to give a more realistic view of how science progresses than is found in textbooks.

With this in mind, *Neural Development *is pleased to announce a forum for such pairs of opinion pieces. The series will focus on aspects of neural development where data are available but a consensus interpretation is lacking. Leading proponents from the apparently conflicting schools of thought will be invited to prepare reviews to be published in parallel.

We begin the series with a pair of articles by Marla Feller [1] and Leo Chalupa [2].

They examine the idea that patterns of spontaneous electrical activity in retinal cells provide instructions required for specific connectivity of retinal axons with their targets in the lateral geniculate nucleus. In essence, Feller argues that several experiments provide convincing evidence that "waves" of retinal activity are determinants of connectivity. In contrast Chalupa argues that the data are equally consistent with the idea that activity is either dispensable or is "permissive" – that is, its presence but not its precise pattern is what matters. The topic is an important one, because this system has been an influential model for analysis of activity-dependent refinement of connectivity, a phenomenon that appears to be widespread in the developing brain.

The rules for submission of these pieces were simple. The authors wrote drafts and exchanged them, then had the opportunity to make revisions if they felt so inclined. In this case, they did not. The manuscripts were then submitted to the journal, and reviewed for fairness by two of the editors. This format has worked well, so we propose to continue it.

We invite readers of Neural Development to add comments, using the 'post a comment' feature available on the full text version of each article. We also hope you will suggest other topics that might benefit from a discourse of this nature. Feel free to nominate yourself or others as authors. Send suggestions to editorial@neuraldevelopment.com.

## References

[B1] Feller MB (2009). Retinal waves are likely to instruct the formation of eye-specific retinogeniculate projections. Neural Development.

[B2] Chalupa LM (2009). Retinal waves are unlikely to instruct the formation of eye-specific retinogeniculate projections. Neural Development.

